# The Luci digital program for reducing dementia risk: efficacy study protocol

**DOI:** 10.1002/alz70860_106996

**Published:** 2025-12-23

**Authors:** Eliana Aubé, Sylvie Belleville, Juliette Guillemont, Simon L Bacon, Michel Boivin, Guylaine Ferland, Kim Lavoie, Guy Paré, Isabelle Lussier

**Affiliations:** ^1^ Lucilab, Montréal, QC, Canada; ^2^ Research Centre, Institut universitaire de gériatrie de Montréal, Montreal, QC, Canada; ^3^ Department of Psychology, Université de Montréal, Montreal, QC, Canada; ^4^ Montreal Behavioural Medicine Centre, Montréal, QC, Canada; ^5^ Department of Health, Kinesiology & Applied Physiology, Montréal, QC, Canada; ^6^ Université de Montréal, Montréal, QC, Canada; ^7^ University of Quebec at Montreal, Montréal, QC, Canada; ^8^ HEC Montreal, Montréal, QC, Canada

## Abstract

**Background:**

Current evidence suggests that a large proportion of dementia cases is associated with lifestyle habits and could thus be prevented. The absence of disease‐modifying treatments highlights the need for widespread access to effective preventive strategies. Luci is a tailored, coach‐supported, fully remote, web‐based, behavioral intervention encouraging healthy lifestyle changes in three domains: diet, physical activity, and cognitive engagement. In prior phases, proof‐of‐concept was established and protocol feasibility demonstrated. Here we describe the protocol of the efficacy trial, which aims to determine if the Luci 24‐week program can reduce the behavioural risk for dementia in middle‐aged to older adults by improving their lifestyle habits.

**Method:**

This 52‐week study will employ a two‐arm, randomized, single‐blind, parallel‐group design. The target population will be cognitively healthy adults aged 50‐75 at risk on at least one of the three intervention domains. 370 participants will be recruited across Canada and randomized to either the Luci intervention or a Waitlist comparison group in a 1:1 ratio. Primary outcomes will be measured with self‐reported behavioural risk questionnaires. Secondary outcomes will comprise cognitive performance and quality of life. Intervention effects will be measured at weeks 12 and 24, with maintenance assessed at week 52.

**Result:**

Based on our prior studies, we defined the primary endpoint as the proportion of participants achieving a clinically significant change in at least one lifestyle domain at week 24. We hypothesize that this proportion will be higher in the Luci group compared to the Waitlist condition, suggesting that Luci can help reduce behavioural risks associated with dementia. Data will be analyzed using logistic regression for the primary endpoint, linear mixed models for secondary outcomes, and structural equation modelling to assess moderators and mediators effects.

**Conclusion:**

A digital program such as Luci has numerous advantages, including reduced barriers to participation (e.g., geographical constraints), increased user convenience (e.g., in‐home participation, flexible scheduling), and lower deployment costs than face‐to‐face delivery. Once validated, it confers the potential for large‐scale implementation as a prevention tool to mitigate the public health burden of dementia.

